# Citations in Wikipedia for understanding research reach

**DOI:** 10.5195/jmla.2024.1730

**Published:** 2024-04-01

**Authors:** Denise Smith, Jennifer McKinnell, Jack Young

**Affiliations:** 1 denisesmith815@gmail.com, Brock University, St. Catharines, ON, Canada; 2 mckinn@mcmaster.ca, Director, Health Sciences Library, McMaster University, Hamilton, ON, Canada; 3 jkyoung@mcmaster.ca, McMaster University, Hamilton, ON, Canada

**Keywords:** Citations, Wikipedia, research reach

## Abstract

**Objective:**

Wikipedia is the most frequently accessed online health information resource and is well positioned as a valuable tool for public health communication and knowledge translation. The authors aimed to explore their institution's health and medical research reach by analyzing its presence in Wikipedia articles.

**Methods:**

In October 2022, a comprehensive database search was constructed in PubMed to retrieve clinical evidence syntheses published by at least one author affiliated with McMaster University from 2017 to 2022, inclusive. Altmetric Explorer was queried using PubMed Identifiers and article titles to access metadata and Wikipedia citation data. 3,582 health evidence syntheses from at least one McMaster University affiliated author were analyzed.

**Results:**

Six percent (n=219) of health evidence syntheses from the authors' institution were cited 568 times in 524 unique Wikipedia articles across 28 different language editions. 45% of citations appeared in English Wikipedia, suggesting a broad global reach for the institutions' research outputs. When adjusted for open access publications, 8% of McMaster University's health evidence syntheses appear in Wikipedia.

**Conclusion:**

Altmetric Explorer is a valuable tool for exploring the reach of an institution's research outputs. Isolating Altmetric data to focus on Wikipedia citations has value for any institution wishing to gain more insight into the global, community-level reach of its contributions to the latest health and medical evidence.

## INTRODUCTION

### Background and Literature Review

McMaster University promotes itself as creating a brighter world through excellence in research across disciplines. The institution takes pride in its commitment “to taking a collaborative approach to improving people's lives, contributing to global knowledge and advancing the health and well-being of the world around us” [1]. McMaster's Health Sciences Library (HSL) supports this mission by facilitating health research excellence, assisting in the exploration and discovery of health information, embracing meaningful community engagement, and providing access to high-quality health information resources in print and online [2]. HSL's research impact services play an integral role in assessing progress towards these “Brighter World” aspirations. Using a combination of traditional metrics (e.g., academic citations; collaboration data) and alternative metrics (e.g., media mentions; Wikipedia citations), the service provides quantitative data and analyses illuminating McMaster's contribution to global health knowledge.

Traditional metrics, like academic citations, can tell us about a publication's influence within the scientific community, but are inadequate for describing its reach outside the academic world [3]. Altmetrics fill this gap by focusing attention on sources that are freely available and widely used by the general public, such as Twitter, news media, and Wikipedia. As such, altmetrics can identify papers that generate interest outside of the academy and point to the potential reach of scholarly research on society at large [4,5]. In addition, papers start receiving attention from altmetric sources as soon as they are published and thus permit more timely assessments of research reach compared to traditional citation-based metrics that can take years to accrue [6]. The Altmetric Explorer database aggregates mentions of academic papers across a wide variety of online information sources and assigns an Altmetric Attention Score (AAS) that represents the level of attention that a particular publication has received [7]. Altmetric also tracks whether a research publication is published using an Open Access publication model (hybrid, green, or gold) or, if it is published in a subscription-based format, requiring readers to pay for access to the content [7].

While the heterogeneous nature of the AAS limits its ability to consistently predict real-world impact [8], it is regularly used as a tool for understanding how information about research travels [9–11]. Consequently, the authors intentionally applied the term reach, instead of impact, for this study, because the goal is to leverage Altmetric Explorer's Wikipedia citation data to gain insight into how far into the community McMaster's health evidence syntheses might reach. The authors propose that citations in Wikipedia articles to McMaster affiliated health-evidence syntheses, could be a potential marker of reach. Altmetric Explorer's data, tracking Wikipedia citations, is an opportunity to learn more about whether McMaster's research outputs are available for consumption in publicly accessible online spaces, like Wikipedia.

The weight with which each mention contributes to a publication's AAS is algorithmically determined based on the mention's reach, which considers the mention's source and author [12]. For example, a tweet authored by a researcher unaffiliated with the publication being shared is weighted more heavily than the same tweet from the article's publisher. Similarly, a citation to the same publication in a Wikipedia article (which has significantly more reach) is weighted more heavily than either of these tweets [13].

Wikipedia is the most frequently accessed health information resource on the Internet [14–17]. In 2013, evidence from a survey indicated that individuals can spend up to 52 hours per year consuming health information on Wikipedia [14]. It is used with greater frequency than the consumer health information web sites libraries might prefer to recommend to their patrons, such as MedlinePlus [16]. While it continues to be stigmatized for its collaborative editing processes [18], the public is accessing Wikipedia's health and medical content to the scale of more than two billion views per year [19]. Most recently, Wikipedia received media attention as a major contributor to the prevention of misinformation during the COVID-19 pandemic [20,21] with the Wikimedia Foundation partnering with the WHO in this regard [22]. Furthermore, Wikipedia was identified by The Lancet as a key player in the amplification of science due to its broad reach [23].

Since its launch in 2001, perceptions of Wikipedia have evolved [24]. Although it is not universally accepted [25], academics, health professionals, and librarians have acknowledged its influence and popularity for the communication of science [26–29] and public health education [23,30–32], despite a limited understanding of how or why readers engage with it [33,34]. Scholarship has explored the benefits associated with including the citation rate on Wikipedia in assessments of the reach of published works [35] and contributing to Wikipedia has continued to gain popularity in medical education [36–40]. Wikipedia also has demonstrated value for the mapping of scientific knowledge [41] while also supporting the open access movement through its preference for summarizing and citing open knowledge sources [42,43]. However, no previous studies of Wikipedia citations as an indicator of the reach of an institution's research outputs were found.

Using Altmetric Explorer to track citations of an institution's publications in Wikipedia, this study aims to gain insight into the reach of a sample of health evidence syntheses published by at least one McMaster University-affiliated author. Using McMaster University as a case example, this study also explores what we can learn about the reach of a research organization through the Wikipedia citations that Altmetric Explorer tracks. Beyond the growing popularity and ubiquity of Wikipedia articles, the authors focused exclusively on citations in Wikipedia because the editorial process requires an element of knowledge translation, has transparent and open process of peer-review, and provides space for community debate to ensure neutrality, accuracy, and verifiability of any contributions made to a Wikipedia article [44].

### Research Questions

To better understand the presence of McMaster University's health and medical research in Wikipedia, the following questions were proposed:

RQ1. What proportion of health evidence syntheses from McMaster University affiliated authors, published between 2017 and 2022, have been cited in Wikipedia?
RQ2. When ranked by AAS, of McMaster University's top 10% highest scoring health evidence syntheses, what proportion are cited in Wikipedia?
RQ3. How many citations to McMaster University's published evidence synthesis outputs from 2017 to 2022 appear in Wikipedia? How many Wikipedia articles do these citations appear in?
RQ4. Is there a relationship between open access publication and a research output's citation in Wikipedia?

## METHODS

In October 2022, a comprehensive database search was constructed in PubMed to retrieve a purposive sample of health evidence syntheses published by at least one author affiliated with McMaster University from 2017 to 2022, inclusive (see Appendix for full search strategy). The decision to search only PubMed was twofold. Firstly, because the authors were interested in using a sample of health evidence syntheses, a large clinical database allowed the authors to comprehensively search for evidence synthesis publications within a discipline-focused resource. Second, PubMed can be publicly accessed and so the author's search strategy to retrieve evidence syntheses can be more easily replicated. Evidence syntheses were selected as the research output to measure because of Wikipedia's guidelines for reliable sources in health and medical articles, which indicate a preference for high-quality secondary sources, including popular methodologies for evidence syntheses such as systematic reviews and meta-analyses [42]. Known within the editing community as WP:MEDRS, these guidelines prioritize high-quality secondary studies (e.g. systematic reviews) published in top-tier medical journals, as determined by Western medical practices [42]. Therefore, not all health and medical research output from the university, for example primary studies, meet the reliability guidelines to be cited in Wikipedia.

The search yielded 4,381 results. 699 results were excluded. Articles were excluded if they were not health related, were an evidence synthesis protocol, original primary research such as a lab experiment or patient study, a white paper, a letter to the editor or editorial, published errata, or if the article focused on evidence synthesis as a topic. Next, Altmetric Explorer was queried using PubMed Identifiers and article titles. Because Altmetric Explorer can be searched using either DOIs or PubMed IDs, the authors searched Altmetric Explorer using the PMIDs retrieved from the PubMed search. The query yielded 97% (n= 3,582) of the articles retrieved from the PubMed search. The authors contacted Altmetric learn why 3% of publications from PubMed were not tracked by Altmetric Explorer but received no response.

The authors exported two data sets to Microsoft Excel from Altmetric Explorer. The first data set, Research Outputs, comprehensively listed every publication that met the search criteria and included a column for the number of times each article had been cited in Wikipedia. The second data set, Wikipedia Mentions, collated the Wikipedia articles that cite at least one of the McMaster University affiliated evidence syntheses, as of October 31, 2022. Both data sheets were used to answer the research questions presented above and gain insight into the reach of the institution's research. Some additional context is required for how the authors approached gathering results for RQ2 and RQ4.

To answer RQ1 the authors employed the COUNTIF command in the Research Outputs dataset spreadsheet to count how many articles had at least one citation in a Wikipedia article. For RQ2, ranking by AAS offered insight into whether the proportion of articles cited in Wikipedia could be affected when the article has received a high AAS. The count of Wikipedia citations would not necessarily impact the AAS in a way that would inherently bias the ranking of articles. This is because “the scoring for Wikipedia articles is static… if a research output is mention[ed] in on Wikipedia post, the score for that paper will increase by 3. However, if a research output is mentioned in more than one Wikipedia post, the score will remain 3” [13]. Therefore, it cannot be assumed that the health evidence syntheses with the highest attention scores are cited in Wikipedia. It can also not necessarily be assumed that being cited in Wikipedia would bias the ranking of research outputs by AAS. That is to say, if all articles only get a score of 3 for being cited in Wikipedia, being cited in Wikipedia would not necessarily bring an article to the top 10% of high-scoring articles.

For RQ4, the authors used a simple random sample (n=347) of all 3,582 of research outputs retrieved to perform a chi-square test for independence in SPSS. The number of results required for a sample that would ensure a 95% confidence interval (n=347) was calculated using a free online Simple Random Sample Calculator [45]. Using RAND() in the data sheet for all 3,582 results, the result set was randomly re-sorted and the top 347 in the list were pulled to make the simple random sample.

## RESULTS

**RQ1. What proportion of health evidence syntheses from McMaster University affiliated authors, published between 2017 and 2022, have been cited in Wikipedia?**

Of the 3,582 health evidence syntheses published between 2017 and 2022 tracked in Altmetric Explorer 6.1% (n=219) were cited in Wikipedia articles at the time of analysis.

**RQ2. When ranked by AAS, of McMaster University's top 10% highest scoring health evidence syntheses, what proportion are cited in Wikipedia?**

Of the top 10% (n=358) of McMaster University's evidence syntheses, ranked by AAS, 29.3% (n=105) were cited in Wikipedia. These 105 articles represented 48% of the 219 outputs cited in Wikipedia and 62.5% (n = 355) of cumulative citations.

**RQ3. How many citations to McMaster University's published evidence synthesis outputs from 2017 to 2022 appear in Wikipedia? How many Wikipedia articles do these citations appear in?**

At the time of analysis there were 568 cumulative citations to McMaster's health evidence syntheses within Wikipedia across 524 unique articles in 29 different language editions (see Table 1). 44.9% (n=255) of the citations were in English Wikipedia.

**Table 1:** Distribution of citations by language fork.

	Wikipedia Language Edition (Wiki prefix)	Citations (n=)	Articles (n=)
1	Arabic (ar)	34	29
2	Bangla (bn)	6	3
3	Catalan (ca)	10	10
4	Czech (cs)	8	5
5	German (de)	22	21
6	Greek (el)	21	14
7	English (en)	255	238
8	Spanish (es)	28	27
9	Farsi (fa)	8	8
10	Finnish (fi)	11	11
11	French (fr)	24	23
12	Hebrew (he)	7	7
13	Hungarian (hu)	8	4
14	Bahasa Indonesian (id)	5	5
15	Italian (it)	15	14
16	Japanese (ja)	15	14
17	Korean (ko)	10	10
18	Dutch (nl)	4	4
19	Polish (pl)	3	3
20	Portuguese (pt)	11	11
21	Romanian (ro)	2	2
22	Russian (ru)	18	18
23	Serbian (sr)	5	5
24	Swedish (sv)	2	2
25	Thai (th)	1	1
26	Turkish (tr)	9	9
27	Ukranian (uk)	3	3
28	Vietnamese (vi)	12	12
29	Zhongwén (zh)	11	11
		568	524

**RQ4. Is there a relationship between open access publication and a research output's citation in Wikipedia?**

Of the 219 health evidence syntheses cited in Wikipedia, 79% (n = 173) were published using an open access model, according to open access classification data within Altmetric Explorer. When the proportion of articles cited in Wikipedia (6.1%) was limited to open access publications, the proportion of articles cited in Wikipedia increased to 8.1%. As outlined in the methods, a random sample (n=347) of all 3,582 health evidence syntheses included in the study was selected and a statistically significant (P < 0.05) relationship between open access evidence syntheses and their presence in Wikipedia was found. (X2(1, N=347) = 4.045, p = 0.044). Therefore, the open access health evidence syntheses included in this study were more likely to be cited in Wikipedia than non-open Access syntheses.

## DISCUSSION

The authors sought to gain initial insights into the reach of McMaster University's health and medicine research publications and the value of using Altmetric Explorer to track Wikipedia citations. This study demonstrates that Altmetric Explorer has some utility for tracking attention gained outside of the academic sphere, specifically to understand the inclusion of McMaster's research in frequently accessed public health information resources. This exploratory study also provides a methodology for future exploration of citations in Wikipedia not necessarily limited to health evidence syntheses at a single institution. Since health and medical librarians regularly participate in the production of evidence syntheses and are also a key resource for researchers wishing to understand their research impact, the findings shared here stand to offer health and medical librarians a methodological approach to gathering an additional dimension in understanding how broadly published health evidence syntheses could be shared.

Our findings show that 6% of health evidence syntheses from McMaster-affiliated authors appear in 524 Wikipedia articles across 29 languages. This provides a useful baseline for understanding one institution's citation activity in Wikipedia.[46] Wikipedia mentions can provide insights not available through traditional citation-metrics, such as the global reach of a work, as represented by McMaster's presence in 29 different language editions of Wikipedia.

The research found that despite 6% of McMaster's health evidence syntheses appearing in Wikipedia, the papers with the highest AAS made up nearly half of the 219 evidence syntheses cited in Wikipedia. These publications accounted for more than half of all Wikipedia citations tracked for this study. In total, nearly 30% of these high scoring publications have been summarized for consumption by the public. In addition to demonstrating the University's reach, data such as this can be related to institutional goals around knowledge translation. Additional investigation into the relationship between AAS and Wikipedia citations is needed for richer insight into the representation of high scoring evidence syntheses in Wikipedia.

With respect to the relationship between open access evidence syntheses and their citation in Wikipedia articles, our finding that the open access evidence syntheses included here are more likely to be cited in Wikipedia than traditionally published (closed) evidence syntheses, is consistent with Wikipedia's well-known preference for verifying its content with citations to open-access materials [42]. This evidence might have utility as libraries continue to strengthen their commitment to promoting open access publishing models. As a potential indicator of mass reach, the relationship between open access publishing models and presence in Wikipedia across multiple languages also has the potential to demonstrate the value of open access publishing.

This study has some limitations. Altmetric tracks Wikipedia citations in real time. Therefore, the evidence syntheses represented in this study are those that were cited in Wikipedia at the time the data was exported. Tracked citations in Wikipedia are not representative of the total number of times a research output has been cited over time. Citations added or deleted after data export are not represented in the results. This does not diminish the results of this exploratory study, but the numbers presented in the results should be considered fluid.

Some McMaster affiliated publications will have overlap with other institutions. While included in estimates of McMaster University's reach, if considered in the context of other institutions' research output, it is important to consider institutional overlap.

Some McMaster researchers have additional affiliations outside of McMaster. Publications in which these authors did not list McMaster as their affiliation were not captured by the PubMed search. This study is also limited to health evidence synthesis and does not represent all knowledge synthesis produced by members of the university.

Given that this was an early exploration of the utility Altmetric Explorer, we only utilized one database (PubMed) to gather a purposive sample of health evidence syntheses from McMaster. Therefore, the collection of evidence syntheses analyzed is not representative of the total output of the University, but rather a snapshot that can be used to inform decision making. Similarly, the authors only analyzed the output from our own University, so the findings can only be considered within the scope of that context. This study does not claim to be generalizable to health evidence syntheses across all institutions, but provides a useful framework for institutions wishing to gain novel insights into their overall research impact.

This study's findings point to a common theme: high quality health and medical information published by academic researchers is made available beyond the boundaries of academia and medical research through being summarized and cited in Wikipedia across multiple articles and in many languages. Understanding the presence of an organization's research in the publicly accessible sources allows for unique insights into the reach of research within society at large. These initial discoveries add dimension to the authors' understanding of the reach of health evidence syntheses from McMaster University affiliated authors.

Medical research is often borne out of a desire to contribute to a healthier society, yet its findings and innovations are regularly produced for a limited audience. Namely, other researchers at other institutions who have both economic and intellectual access to the material. If citations alone are measured, there is a risk of measuring the activity within a closed system.

With the advent of Altmetric Explorer, Wikipedia mentions are now just as easy to track as academic citations and offer a proxy for understanding societal reach of scholarly work. The public is becoming more proficient at consuming health information from home and understanding the presence of a research organization's output in Wikipedia articles has the potential to add dimension to the story. By isolating Altmetric tracking data to Wikipedia mentions, the authors gained valuable insight into the broad global reach of McMaster's health evidence syntheses and identified opportunities for more thorough exploration of Altmetric data sets and Wikipedia mentions.

## DATA AVAILABILITY STATEMENT

Although Altmetric Explorer provides metrics on an article-by-article data, aggregate data pulled from a suite of articles in Altmetrics cannot be made publicly available. This is a feature of the proprietary Altmetric Explorer product. Therefore, the raw data associated with this article cannot be made publicly available because the data retrieved from Altmetric Explorer is owned by Altmetric. The authors' complete PubMed search strategy, used to yield health evidence syntheses from McMaster University-affiliated authors, is supplied in Appendix A.

## AUTHOR CONTRIBUTIONS

Denise Smith: conceptualization, data curation; formal analysis, methodology, project administration, visualization, writing-original draft, writing – review & editing; Jack Young: data curation, methodology, writing - original draft, writing – review & editing; Jennifer McKinnell: conceptualization, data curation; methodology, writing - original draft, writing – review & editing.

## SUPPLEMENTAL FILES

**Appendix A:** Search Strategy
