# Supplementary material for: Citations in Wikipedia for understanding research reach
Source: J Med Libr Assoc. 2024 May 22;112(2):88–94. doi: 10.5195/jmla.2024.1730 (PMC11305477; doi:10.5195/jmla.2024.1730)
Supplement: Supplementary file 1 — Appendix A: Search Strategy [file jmla-112-2-88-s01.docx]

**Search Strategy**

Source: PubMed

Date run: October 31, 2022

Search:

("meta-analysis"[Publication Type] OR systematic review[Publication Type]) OR (umbrella[Title/Abstract] OR "systematic*"[Title/Abstract] OR "scoping"[Title/Abstract] OR "metaanalys*"[Title/Abstract] OR "meta analys*"[Title/Abstract] OR "meta analys*"[Title/Abstract] OR "evidence synthesis"[Title/Abstract]) AND "mcmaster"[Affiliation] AND 2017/01/01:2022/12/31[Date - Publication]
